# Morphine Attenuates Testosterone Response to Central Injection of Kisspeptin in Male Rats

**Published:** 2014-07-08

**Authors:** Fariba Mahmoudi, Homayoun Khazali, Mahyar Janahmadi

**Affiliations:** 1 Department of Physiology, Faculty of Biological Sciences, Shahid Beheshti University, Tehran, Iran; 2 Neurophysiology Reseaech Center and Department of Physiology, Medical School, Shahid Beheshti University of Medical Science,Tehran, Iran

**Keywords:** Kisspeptin, Morphine, Naloxone, Testosterone

## Abstract

**Background:**

Kisspeptin and naloxone stimulate the reproductive axis while morphine inhibits its function. We have investigated the effect of central injection of kisspeptin-10 on mean plasma testosterone concentration in morphine or naloxone pretreated rats.

**Materials and Methods:**

In this experimental study, 60 male Wistar rats that were divid- ed into 12 groups (n=5 per group) received saline, kisspeptin (1 nmol, ICV), naloxone (2 mg/kg, subcutaneously), morphine (5 or 10 mg/kg, sc) or co-administrations of kisspeptin, morphine and naloxone at 09:00 - 09:30. In the co-administrated groups, kisspeptin was injected 15 minutes following morphine or naloxone injections. Blood samples were collected 60 minutes following injections via the tail vein. Plasma testosterone concentration was measured by a rat testosterone ELISA kit.

**Results:**

Central injection of kisspeptin or subcutaneous injection of naloxone significantly increased the mean plasma testosterone concentration compared to saline while subcutaneous injections of different doses of morphine (5 or 10 mg/kg) significantly decreased testosterone compared to saline. The results revealed that morphine significantly attenuated the testosterone increase after kisspeptin injection compared to kisspeptin while a stimulatory additive effect was observed in the kisspeptin/naloxone group compared to either naloxone or kisspeptin.

**Conclusion:**

Morphine and kisspeptin systems may interact with each other to control the hypothalamic-pituitary-gonadal (HPG) axis.

## Introduction

Narcotics and opioid peptides are known to inhibit the activity of the hypothalamic-pituitarygonadal (HPG) axis in rodents, ruminants and humans. Central or peripheral injections of morphine or β-endorphin significantly decrease mean plasma luteinizing hormone (LH) and circulating gonadal steroids mainly via binding to opioid μ-type receptors (1-6). Naloxone, an opioid receptor antagonist competitively inhibits the effects of opioids on the HPG axis activity; it increases the LH and gonadal hormone secretions in males and females of different species (7-9). It has been demonstrated that the inhibitory effects of opioids on the HPG axis is mediated mainly via hypothalamic gonadotropinreleasing hormone (GnRH) rather than direct effects on pituitary gonadotrophs (9-16). Opiates are increasingly consumed as drugs of abuse or drugs for pain management. Opiate addiction or opioid therapy for the management of chronic cancer pain affects the gonadal functions and induces hypogonadism, amenorrhea and infertility (17, 18).

Kisspeptin-54 is the product of the *KiSS1* gene and further proteolytical cleaving of it gives rise to shorter products namely kisspeptin-14, kisspeptin-13 and kisspeptin-10. All kisspeptins share a common COOH-terminal decapeptide and they have similar affinity to the G protein-coupled receptor, GPR54 (19-21). Recently, it has been revealed that the kisspeptin/GPR54 signaling system plays a critical role in regulation of the HPG axis. The GPR54 receptor is necessary for normal pubertal development and sexual function (22). It has been shown that dysfunctional or mutations in the gene encoding the GPR54 receptor cause hypogonadotropic hypogonadism and pubertal delay in humans and rodents (23, 24). The kisspeptin action on the HPG axis is esteroid dependent so that expression of *KiSS1* mRNA during the estrus phase is less than other phases of the cycle. Its expression peaks during the evening proestrus where circulating levels of LH begin to rise rapidly and peak in the evening, resulting in ovulation (25-27). It has been shown that the majority of GnRH neurons express the GPR54 receptor and central or peripheral administration of kisspeptin potently stimulates the HPG axis. Central or peripheral injection of the GPR54 receptor antagonist, peptide 234 significantly blocks the stimulatory effect of kisspeptin on HPG axis activity (28). Accumulating evidence has shown that endogenous opioid peptides and opiates (e.g., morphine) exert their inhibitory effects on the HPG axis via different interneurons (29, 30). The arcuate nucleus (ARC) kisspeptin/neurokinin B (NKB)/dynorphin (KNDy) neurons play a role in estrogen negative feedback action on pulsatile GnRH/LH release (31-35). These recent studies have shown that the interaction between hypothalamic kisspeptin and dynorphin play a role in controlling the HPG axis, however there is no report about the interaction of morphine (the main opiate in HPG axis activity) and the kisspeptin signaling system on the HPG axis thus far. In the present study we aim to investigate the effects of kisspeptin-10 on mean plasma testosterone concentration in morphine or naloxone-treated male rats with the intent to determine if kisspeptin can be a suitable therapeutic target for pharmacological intervention of reproductive dysfunctions caused by opiates.

## Materials and Methods

### Animals

In the present experimental study, male Wistar rats (n=60) that weighed 230-250 g were obtained from the Center of Neuroscience Research at Shahid Beheshti University (Iran) and housed individually in cages under controlled temperature (22 ± 2˚C) and light (12 hour light/dark cycle, lights on at 0700 hours). Animals had continual free access to food and water. All procedures for the maintenance and the use of the experimental animals received the approval of the Ethical Committee of the Neuroscience Research Center at Shahid Beheshti University of Medical Sciences (Tehran, Iran).

### Intra-cerebral ventricular (ICV) cannulation and injections

Animal surgery procedures and handling were carried out as previously described (36). Animals were anesthetized by intraperitoneal (IP) injections of a mixture of ketamine (80 mg/kg bw) and xylazine (10 mg/kg bw). For central injections, a 22-gauge stainless cannulae was implanted in the third cerebral ventricle according to the coordinates of the Paxinos and Watson Atlas (AP=- 2.3, ML=0.0, DV=6.5). The cannula was secured to the skull with three stainless steel screws and dental cement. The animals were kept in individual cages.

After a one-week recovery period, the 60 rats in 12 groups of n=5 per group received either saline (3 μl)/ saline (200 μl), kisspeptin (1 nmol/3 μl)/ saline (200 μl), saline (3 μl)/ morphine (5 mg/kg, 200 μl), saline (3 μl)/ morphine (10 mg/kg, 200 μl), saline (3 μl)/ naloxone (2 mg/kg, 200 μl), saline (3 μl)/ naloxone (2 mg/kg, 100 μl) + morphine (5 mg/kg, 100 μl), saline (3 μl)/ naloxone (2 mg/kg, 100 μl) + morphine (10 mg/kg, 100 μl), kisspeotin (1 nmol/3 μl)/ morphine (5 mg/kg, 200 μl), kisspeptin (1 nmol/3 μl)/ morphine (10 mg/kg, 200 μl), kisspeotin (1 nmol/3 μl)/ naloxone (2 mg/kg, 200 μl), kisspeotin (1 nmol/3 μl)/ naloxone (2 mg/kg, 100 μl) + morphine (5 mg/kg, 100 μl), kisspeotin (1 nmol/3 μl)/ naloxone (2 mg/kg, 100 μl) + morphine (10 mg/kg, 100 μl). kisspeptin was injected via third cerebral ventricle and morphine or naloxone were injected subcutaneously.

For ICV injection, kisspeptin-10 (Ana Spec Co., USA) was dissolved in DMSO and injected with a 27-gauge stainless steel injector that protruded 0.5 mm beyond the cannula, and was connected to a Hamilton microsyringe by PE-20 tubing at 9-9:30 AM. For subcutaneous injection, morphine sulfate (Temad Co., Iran) and naloxone hydrochloride (Tolid Daru Co., Iran) were dissolved in distilled water and injected by an insulin syringe at 9-9:30 AM. In co-administrated groups, either morphine or naloxone injections were administered 15 minutes before the kisspeptin-10 injections.

### Hormone assays and statistical analyses

We collected 0.5 cc of blood at 60 minutes following the injections via the tail vein (37, 38). Heparin was used to the samples to prevent clotting. Blood samples were immediately centrifuged for 15 minutes at 3000 rpm and the plasma stored at -20˚C until assayed for testosterone concentrations. Plasma testosterone concentration was measured by using a rat testosterone kit and ELISA. The kit had a sensitivity of 0.06 ng/ml, intra-assay of 6.61% and inter-assay of 9.32%. The results are presented as mean ± SEM. The data were analyzed by the one-way ANOVA test followed by post hoc Tukey’s test and SPSS software (version 16). In all cases, significance was defined by p<0.05.

## Results

The results showed that injection of kisspeptin/saline significantly increased the mean plasma testosterone concentration compared to saline/saline group. Injection of saline/morphine (5 or 10 mg/kg) significantly decreased the mean plasma testosterone concentration compared to the saline/saline group. A significant difference was not observed between the effects of 5 mg/kg and 10 mg/kg of morphine on mean testosterone concentration . Mean plasma testosterone concentration significantly increased following saline/naloxone injection compared to the saline/saline or saline/morphine (5 or 10 mg/kg) groups.

The results also revealed that in the naloxone pretreated groups, morphine did not significantly decrease testosterone concentration compared to the saline/morphine (5 or 10 mg/kg) groups. We did not observe a significant difference between the effects of morphine on testosterone concentration in the naloxone pretreatment groups. The mean plasma testosterone concentration significantly decreased following co-administration of morphine (5 or 10 mg/kg)/kisspeptin compared to the kisspeptin/saline group (Fig ure 1, Table 1). The mean plasma testosterone concentration significantly increased after co-administration of naloxone/kisspeptin compared to the saline/naloxone injected group. Also, the testosterone concentration increased in the kisspeptin/naloxone group compared to the kisspeptin/saline group but this increase was not statistically significant.

**Fig 1:**
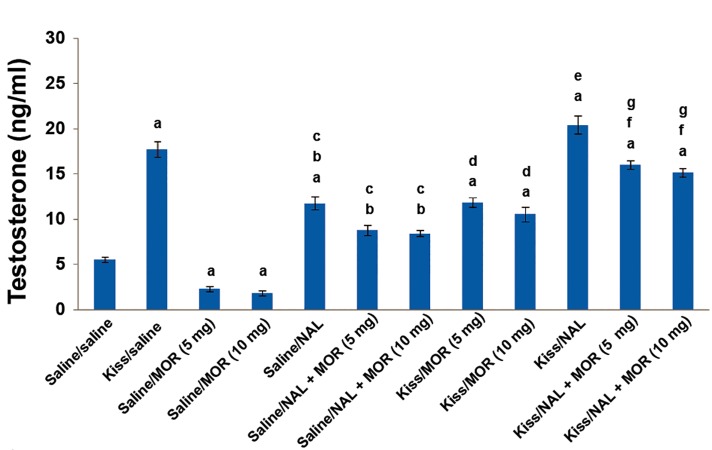
Effects of kisspeptin, morphine, naloxone or co-administration of kisspeptin, morphine and naloxone on mean plasma testosterone concentration in male Wistar rats. Significant differences are indicated by letters. a; Compared to saline/saline group, b; Compared to saline/5 mg/kg morphine group, c; Compared to saline/10 mg/kg morphine group, d; Compared to kisspeptin/saline group, e; Compared to saline/naloxone group, f; Compared to kisspeptin/5 mg/kg morphine group, g; Compared to kisspeptin/10 mg/kg morphine group (data presented as mean ± SEM; p<0.05; n=5 per group), NAL; Naloxone and MOR; Morphine.

The results also showed that the mean plasma testosterone concentration significantly increased after co-administration of kisspeptin/naloxone + morphine (5 or 10 mg/kg) compared to kisspeptin/morphine. However a significant difference was not observed between the effects of co-administration of kisspeptin/naloxone + 5 mg/kg morphine and kisspeptin/naloxone + 10 mg/kg morphine on testosterone concentration. A significant difference was not observed between the effects of co-administration of kisspeptin/naloxone + morphine (5 or 10 mg/kg) on testosterone concentration compared to kisspeptin/saline (Fig 1, Table 1).

**Table 1:** Effects of kisspeptin, morphine, naloxone or co- administration of kisspeptin, morphine and naloxone on mean plasma testosterone concentration (data presented mean ± SEM, P<0.05, n=5 per groups).


Treatments	Testosterone(ng/ml)

**saline/saline**	5.81 ± 0.28
**1 nmol kisspeptin/saline**	17.76± 0.85
**saline/5mg/kg morphine**	2.33 ± 0.26
**saline/10mg/kg morphine**	1.85 ± 0.3
**saline/2mg/kg naloxone**	11.7± .73
**saline/ 2mg/kg naloxone+ 5mg/kg morphine**	8.81± 0.44
**Saline/ 2mg/kg naloxone+ 10mg/kg morphine**	8.43± 0.33
**1 nmol kisspeptin/5mg/kg morphine**	11.89± 0.55
**1 nmol kisspeptin/10mg/kg morphine**	10.30± 0.75
**1 nmol kisspeptin/ 2mg/kg naloxone**	20.43± 0.99
**1 nmol kisspeptin/ 2mg/kg naloxone+ 5mg/kg morphine**	16.05± 0.45
**1 nmol kisspeptin/2mg/kg naloxone+ 10mg/kg morphine**	15.17± 0.47


## Discussion

In the present study, we chose the kisspeptin, naloxone and morphine doses based on previous studies which reported their respective stimulatory or inhibitory effects on the HPG axis (5-8, 37, 38). We first examined whether the selected doses would be effective in our laboratory. The results showed that subcutaneous injection of 2 mg/kg naloxone significantly increased the mean plasma testosterone concentration compared to saline while subcutaneous injections of different doses of morphine (5 or 10 mg/kg) significantly decreased mean plasma testosterone compared to saline. These results were consistent with previous studies which reported stimulatory effects of naloxone and inhibitory effects of opioid peptides on the HPG axis (1-9). However the precise mechanism of the inhibitory effects of opioids on the reproductive axis is controversial but majority of the previous studies have demonstrated that endogenous opioids or exogenous opiates (e.g., morphine) and their antagonists appear to influence the release of LH from the pituitary and subsequently gonadal steroid hormones. It is believed that hypothalamic neurotransmitters, particularly down regulation of noradrenergic or up regulation of GABAergic neurons, play an important role in relaying the indirect effects of opioids and their antagonists on hypothalamic GnRH-producing neurons (29, 30). Additionally, the dose of kisspeptin in the present study has significantly increased the mean plasma testosterone concentration compared to saline. This result agrees with previous studies which have demonstrated that kisspeptin is a key component of reproduction that directly stimulates the HPG axis. Both central and peripheral administration of kisspeptin can increase plasma LH, FSH and total testosterone (37-40).

However previous studies have suggested that opioid peptides exert their inhibitory effects on the HPG axis via different interneurons and there is an interaction between kisspeptin and dynorphin in controlling the HPG axis but there is no report about the interaction of morphine and the kisspeptin/GPR54 signaling system on the HPG axis thus far. In the present study, we have investigated the effects of a central injection of kisspeptin on mean plasma testosterone concentration following morphine or naloxone injections. The results showed that morphine significantly attenuated testosterone response to the kisspeptin injection compared to the kisspeptin alone group while a stimulatory additive effect was observed in the kisspeptin/naloxone group compared to only naloxone. In the present study, for the first time, the effect of an interaction between morphine and kisspeptin has been investigated on the reproductive axis. No previous studies exist to compare these results. However morphine may play a partial role in the rate of kisspeptin/GPR54 signaling system activity. Recently it has been established that some important factors involving the control of sexual function that include steroid hormones, fasting or ghrelin exert their inhibitory effects on the HPG axis via down regulation of the kisspeptin/GPR54 signaling system. Ghrelin injection results in a significant decrease in *KiSS1* gene mRNA level in the brain (41-43). It has been revealed that leptin or even photoperiod (in seasonal animals) exerts stimulatory effects on the reproductive axis via up regulation of kisspeptin gene expression (44-46). Most recent studies have reported that the kisspeptin signaling system is involved in the pathways of reproductive factors. In the current study we have demonstrated that injection of morphine decreased the stimulatory effect of kisspeptin on testosterone secretion, therefore one can expect that morphine may interact with the kisspeptin system to control the HPG axis. In future studies we intend to examine the effects of injections of morphine, naloxone or their simultaneous injections on *KiSS1* or *KiSS1r* (GPR54 receptor) gene expression level or other inhibitory or stimulatory factors involved in reproduction such as ghrelin and leptin in order to understand the possible direct or indirect interaction of opioids with the kisspeptin system in controlling HPG axis activity.

## Conclusion

The results of the present study showed that central injection of 1 nmol kisspeptin or subcutaneous injection of 2 mg/kg naloxone significantly increased mean plasma testosterone concentrations compared to saline while subcutaneous injection of different doses of morphine (5 or 10 mg/kg) significantly decreased mean plasma testosterone compared to saline. Morphine significantly attenuated testosterone secretion after kisspeptin injection compared to the kisspeptin only group while a stimulatory additive effect was observed in the kisspeptin/naloxone group compared to only naloxone or kisspeptin. Thus the morphine and kisspeptin systems might interact with one another to control the HPG axis.
